# Carbon Material-Based Aerogels for Gas Adsorption: Fabrication, Structure Design, Functional Tailoring, and Applications

**DOI:** 10.3390/nano12183172

**Published:** 2022-09-13

**Authors:** Lianming Zhang, Yu Lei, Peng He, Hao Wu, Lei Guo, Gang Wei

**Affiliations:** 1 Engineering Research Center of Green Process, School of Resources and Environmental Engineering, Shandong Agriculture and Engineering University, Jinan 250100, China; 2 Institute of Biomedical Engineering, College of Life Science, Qingdao University, Qingdao 266071, China; 3 College of Chemistry and Chemical Engineering, Qingdao University, Qingdao 266071, China

**Keywords:** carbon materials, aerogels, hybrid materials, functional tailoring, gas adsorption

## Abstract

Carbon material-based aerogels (CMBAs) have three-dimensional porous structure, high specific surface area, low density, high thermal stability, good electric conductivity, and abundant surface-active sites, and, therefore, have shown great application potential in energy storage, environmental remediation, electrochemical catalysis, biomedicine, analytical science, electronic devices, and others. In this work, we present recent progress on the fabrication, structural design, functional tailoring, and gas adsorption applications of CMBAs, which are prepared by precursor materials, such as polymer-derived carbon, carbon nanotubes, carbon nanofibers, graphene, graphene-like carbides, fullerenes, and carbon dots. To achieve this aim, first we introduce the fabrication methods of various aerogels, and, then, discuss the strategies for regulating the structures of CMBAs by adjusting the porosity and periodicity. In addition, the hybridization of CMBAs with other nanomaterials for enhanced properties and functions is demonstrated and discussed through presenting the synthesis processes of various CMBAs. After that, the adsorption performances and mechanisms of functional CMBAs towards CO_2_, CO, H_2_S, H_2_, and organic gases are analyzed in detail. Finally, we provide our own viewpoints on the possible development directions and prospects of this promising research topic. We believe this work is valuable for readers to understand the synthesis methods and functional tailoring of CMBAs, and, meanwhile, to promote the applications of CMBAs in environmental analysis and safety monitoring of harmful gases.

## 1. Introduction

Aerogels are types of solid materials with three-dimensional (3D) porous, interconnected network structures composed of various precursor materials, such as colloidal particles, nanofibers/nanowires, and nanosheets, through both chemical and physical cross-linking [1,2,3]. This kind of specific structure of aerogels ensures they have high specific surface area, low density, and high porosity. Various polymers, inorganic nanomaterials, and biomass have been utilized for the preparation of aerogels, with the desired structures and functions for wide applications, from materials science to energy science, environmental science, biomedicine, analytical science, and many others [4].

With the development of materials science and nanotechnology, various carbon materials, such as biomass-derived carbon [5], carbon nanotubes (CNTs) [6], polymer-based carbon nanofibers (CNFs) [7], graphene [8], and graphene-like carbides (such as SiC and Ti_3_C_2_T_x_) [9,10] have been utilized for the fabrication of carbon material-based aerogels (CMBAs). Similar to traditional fabrication techniques of both polymer and inorganic aerogels, CMBAs can also be firstly prepared through the formation of 3D hydrogels., Subsequent drying (like both freeze-drying and room-temperature drying) and thermal reduction or carbonization can then create 3D hydrogels by sol–gel polymerization, hydrothermal reaction, self-assembly, electrospinning, 3D-printing, and templated synthesis [11]. Compared to traditional polymeric, inorganic, and biomass material-based aerogels, CMBAs have revealed the advantages of higher thermal stability and electric conductivity, as well as higher surface area, lower density, and more actives sites, enabling CMBAs to be excellent candidates for the fabrication of multifunctional materials in various applications, including electrochemical batteries, supercapacitors, wearable devices, electromagnetic interference shields, thermal insulators, and environmental adsorbents. For instance, Wang et al. reported the modification of ZIF-8 with the biomass, agarose, as carbon resource for the fabrication of CMBAs via pyrolysis [12]. The formed CMBAs exhibited 3D porous structure and high potential for the adsorption of organic pollutants. Huang et al. demonstrated the synthesis of 3D graphene aerogels (GAs) with cellular structure, which could be effectively used for the adsorption of electromagnetic waves [13]. Zhang and co-workers presented the preparation of neat 3D C_3_N_4_ aerogels through room-temperature polymerization, which exhibited high thermal stability and photo-reactivity [14].

Previously, a lot of reviews on the synthesis and applications of CMBAs have been released. For example, Lee et al. summarized recent advances in the fabrication of CMBAs for energy storage, catalysis, water purification, thermal insulators, and gas storage [15]. Hu et al. provided their viewpoints on how to enhance the mechanical properties of CMBAs and promote their applications in insulation [16]. Huang and co-workers demonstrated the progress in applying bacterial cellulose for the fabrication of CMBAs, which revealed potential applications in energy storage, microwave attenuation, and specific adsorption [17]. Gan and co-workers presented a minireview on the synthesis of carbon aerogels (CAs) for environmental clean-up, through CA-based adsorption and catalysis [18]. Various drying and carbonization methods for CAs, as well as their applications in oil/water separation, removal of organic compounds and CO_2_, and catalytic degradation of hazardous compounds were introduced. In a recent article, Keshavarz and colleagues provided a comprehensive review on the synthesis of aerogels (also including CMBAs) for CO_2_ adsorption [19], in which the case studies on the CO_2_ adsorption performance, kinetics, and thermodynamics by aerogels were introduced and discussed in great detail. Studying these review articles, we noticed that the progress on the fabrication of CMBAs for gas adsorption has not been comprehensively reported.

Herein, in this manuscript, we present a comprehensive review on the fabrication, functional regulation, and gas adsorption applications of CMBAs that have been reported in the period 2016–2022. To achieve this aim, first we introduce the synthesis methods of aerogels, including the fabrication of hydrogels using the following methods: various types of self-assembly, hydrothermal synthesis, electrospinning, 3D printing, and others. We also introduce the subsequent drying processes. Then, the preparation of CMBAs from different carbon resources, such as polymers, carbon nanotubes (CNTs), carbon nanofibers (CNFs), graphene, and carbides, are summarized. After that, the fabricated CMBAs for the adsorption of CO_2_, CO, H_2_S, H_2_, and organic gases are analyzed and discussed. Finally, the challenges and potential research outlooks are discussed. We believe this review could fill in the gap to present comprehensive applications of CMBAs. In addition, it will be valuable for inspiring advanced applications of CMBAs through the design and functionalization of precursor materials.

## 2. Synthesis and Structural/Functional Tailoring of Aerogels

In this section, the synthesis of various aerogels, and their structural, as well as functional, tailoring are introduced and discussed.

### 2.1. Synthesis of Aerogels

The preparation of 3D structures can be achieved by various methods, including electrospinning, hydrothermal synthesis, self-assembly, and 3D printing, and the formed 3D materials can be freeze-dried and atmospherically dried to fabricate aerogels.

**Figure 1:**
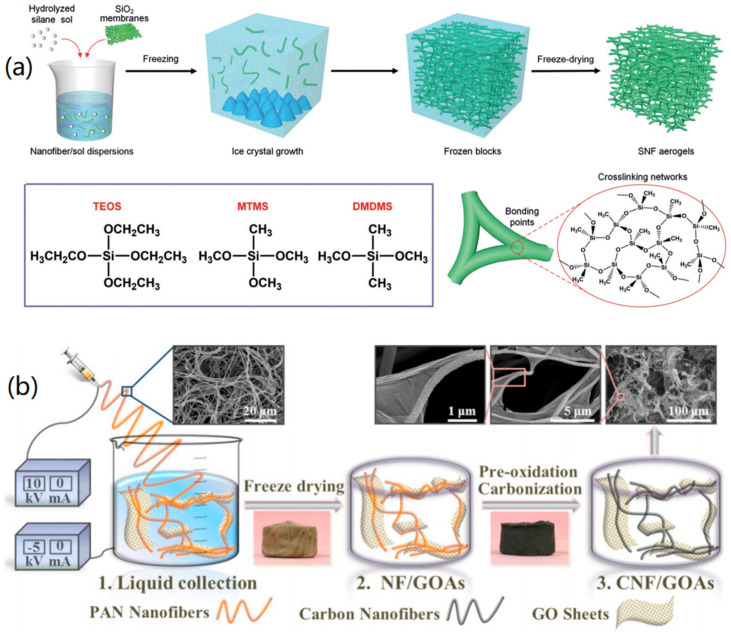
Fabrication of aerogels: (**a**) Freezing-mediated growth and freeze-drying fabrication of SNF aerogels. Reprinted with permission from Ref. [20], Copyright 2020, Wiley VCH. (**b**) Electrospinning and freeze-drying promoted fabrication of CNF/GO hybrid aerogels. Reprinted with permission from Ref. [21], Copyright 2019, Elsevier.

For instance, Wang et al. prepared silica nanofiber (SNF) aerogels with rush-like biomimetic nanofiber frameworks by an in situ synthesis strategy [20]. As shown in Figure 1a, the hydrolyzed silane sol is synthesized by combining three silane coupling agents, ethyl orthosilicate, trimethoxymethylsilane, and dimethoxydimethylsilane that are dissolved in water/tert-butanol/oxalic acid mixture. Then, the electrospun flexible SNFs were homogenized in a silane hydrolysate to obtain the nanofiber/sol dispersion, which was then frozen in liquid nitrogen and freeze-dried to form SNF aerogels. When the dispersion freezes, solid-phase components are expelled from the ice crystals growing in all directions, due to phase separation, thus, allowing the formation of a continuous skeletal network between the crystals. Meanwhile, uniform silane-hydrolyzed sol would wrap around the surface of the nanofibers and become nodes for bonding adjacent fibers. Subsequent freeze-drying allowed the SNF aerogel to obtain an isotropic skeletal structure similar to that of rush pith. The uniform biomimetic framework formed by super-elastic SNFs can dissipate external stress to a large extent, thereby improving the mechanical properties of aerogels. Therefore, the obtained SNF aerogels have ultra-low density (>0.25 mg cm^−3^), ultra-high porosity (99.989%), extremely low thermal conductivity (24 mW m^−1^ K^−1^), and excellent fire resistance.

Zheng et al. reported an ultralight CA that was assembled from CNFs and graphene oxide (GO) through a liquid-assisted collection-electrospinning technique [21]. As shown in Figure 1b, electrospun polyacrylonitrile (PAN) nanofibers were directly collected in the GO aqueous dispersion. During this process, PAN nanofibers were assembled with GO sheets to form an open porous network. More importantly, the non-solvent spontaneously entered into the inner space of the fiber network, increasing the 3D space between nanofibers, thus, forming a uniform continuous nanofiber skeleton. Ultralight 3D CNF/GO aerogels (CNFs/GOAs) with high compressibility were obtained after freeze-drying and high-temperature carbonization. The whole process does not require mechanical dispersion and chemical cross-linking, and can greatly reduce the amount of GO sheets. This method is facile for the production of PNF/carbon hybrid materials and their aerogels.

Wang and colleagues synthesized ultralight, porous ZIF-8/AG-CAs by a simple and sustainable method using cheap and readily available biomass agarose (AG) as a carbon precursor [12]. Firstly, ZIF-8 was mixed with AG to form ZIF-8/AG hydrogels, which were freeze-dried and carbonized to synthesize ZIF-8/AG-CAs. The molecular sieves of ZIF-8 and AG, with high specific surface area and high nitrogen content, had a good synergistic effect on the construction of the gel network, in which ZIF-8 helped to maintain the two-dimensional (2D) sheet structure of AG, and AG promoted the extension of ZIF-8. Therefore, the formed aerogels exhibited a highly interconnected porous 3D structure.

In addition to freeze-drying, supercritical drying can also be utilized for the fabrication of various aerogels [4,22]. When the temperature and pressure are above critical levels, the liquid in the as-prepared hydrogels can transform into a gas directly without any phase transition, and the structure of the formed aerogels is not be affected by the capillary forces, which is different from the freeze-drying method. For instance, Yue and co-workers reported the synthesis of N-doped GAs through supercritical CO_2_ drying [23]. The formed GAs exhibited low thermal conductivity and low bulk density, and could be potentially utilized for the production of thermal insulation systems. Water/CO_2_, ethanol, methanol, and alcohols can be used as solvents for supercritical drying to form aerogels. Cheng et al. reported the synthesis of GAs via supercritical ethanol drying and subsequent high temperature reduction [24]. The created GAs revealed enhanced mechanical, electric, and thermal properties, and could be useful for the fabrication of flexible batteries and insulation devices.

Aerogels can also be fabricated by the atmospheric drying method. Inspired by damselflies forming wings in the natural environment, Han et al. developed a generalized atmospheric green drying method for producing aerogels by using sodium bicarbonate solution as a non-low surface tension (LST) solvent [25]. Instead of using the organic solvent of LST to suppress gel network collapse during drying, this approach used sodium bicarbonate solution to generate in situ CO_2_-supporting pores. Compared with supercritical drying and freeze-drying methods, this method greatly reduced energy consumption, time, and cost of aerogel production.

However, the structure and gas adsorption properties of CMBAs are affected by the drying methods used. For example, ambient pressure drying is easy for operation with low energy consumption, but it is not suitable for the preparation of some soft aerogels, as the capillary forces and adhesion forces at the liquid–air interfaces destroy the network structure of the aerogels. In the freeze-drying method, capillary forces cannot affect the structure of formed aerogels, but the freezing process is very crucial for tailoring the pore size and network structure of aerogels. If the formed ice crystals are too big, the pore size of aerogels is large, which induces shrinking of the aerogel structure. Therefore, both ambient pressure drying and freeze-drying have undesired effects on the structure and properties of formed aerogels, and more efforts should be made to modify the drying process. Supercritical drying can make aerogels with the desired structure; however, it involves high cost and a long timescale for the fabrication of aerogels, which limits its practical applications for the preparation of large-scale CMBAs.

Mezzenga and co-workers prepared fibril@silica core-shell structural aerogels by mixing amyloid fibrils (β-lactoglobulin and lysozyme fibrils) with the silica precursor tetraethyl orthosilicate (TEOS) [26]. During this synthesis process, the electrostatic attraction between amyloid fibrils and silica is the main driving force for the formation of a uniform fibril@silica core-shell structure. The specific surface area of fibril@silica core-shell nanostructured aerogels formed after solvent exchange, supercritical CO_2_ drying, and calcination. The synthesis method of this material facilitates expansion to the rational construction of dual networks of amyloid fibrils and hydrophilic polymers.

Xu et al. used a two-step method combining hydrothermal synthesis and in situ pyrolysis to prepare ultrafine Ni nanocrystal-supported magnetic graphene aerogel (GA@Ni) [27]. Using nickel acetylacetonate (PPNi) as the nickel source, mixed polyvinyl alcohol (PVA) and GO suspensions were hydrothermally reacted at high temperature to form the hydrogel, which was then freeze-dried and calcined at high temperature to obtain bare Ni particle-loaded GA. The resulting GA@Ni possessed superparamagnetic properties and strong microwave absorption properties.

### 2.2. Structural Tailoring

In the preparation of aerogels, solvent exchange is a relatively common method, and the use of different solvents often has different effects on the structure of aerogels. In order to optimize the lithium storage and stability of SiOC aerogels, Sasikumar et al. obtained SIOC aerogels with different structures by changing the synthesis and processing conditions [28]. The obtained results demonstrated that the aerogels synthesized in cyclohexane were significantly different from those synthesized in acetone, in terms of linear shrinkage and density. For instance, the aerogels synthesized in acetone had large porosity, which was beneficial to improve the initial capacity of the capacitor, while lower porosity would improve the stability of the synthesized aerogels. In the report of Teo et al. [29], the influence of the solvent during the synthesis of polyimide (PI) aerogel was explored through using single or mixed solvents to cross-link the PI gel network, with subsequent supercritical drying. It was found that the gelation time of PI prepared in 100% N,N-dimethylformamide (DMF) was only 6.5 min, while the gelation times of PI in 1-methyl-2-pyrrolidone (NMP) and N,N-dimethylacetamide (DMAc) were 112 and 109 min, respectively. Correspondingly, the chain sizes of the fibrous structures and the specific surface areas of the synthesized aerogels obtained in these different solvents were also different. The aerogels obtained in DMF have the shortest gelation time, smallest chain diameter, and the largest specific surface area, while the aerogels created with NMP and DMAc revealed nanofibrous polymer chains with larger diameters, which, in turn, adversely affected the specific surface area and pore size of the formed aerogels.

Similarly, Lee et al. reported a method capable of controlling the structure of PI aerogels [30]. Solvent exchange by a solvent with a stronger affinity for PI produced aerogels with smaller shrinkage, and the porosity was inversely proportional to the shrinkage rate. The smaller the shrinkage of the aerogel, the greater the porosity. In addition, the affinity of the solvent also affects the shape of the pores. When a solvent with poorer affinity for PI was added into the hydrogel system, a larger-scale phase separation occurred, resulting in a larger pore size. Mi et al. developed a facile and efficient method for the preparation of regenerated cellulose aerogels, using a high concentration of 1-allyl-3-methylimidazolium chloride (AMIMCl) aqueous solution, instead of the traditional aqueous solution, as a regenerator, following treatment through the solvent exchange method with ethanol and supercritical drying [31]. The use of AMIMCl in the regeneration process changed the gelation behavior of cellulose, resulting in a uniform nanoporous structure with excellent compressibility, low density, and low thermal conductivity.

**Figure 2:**
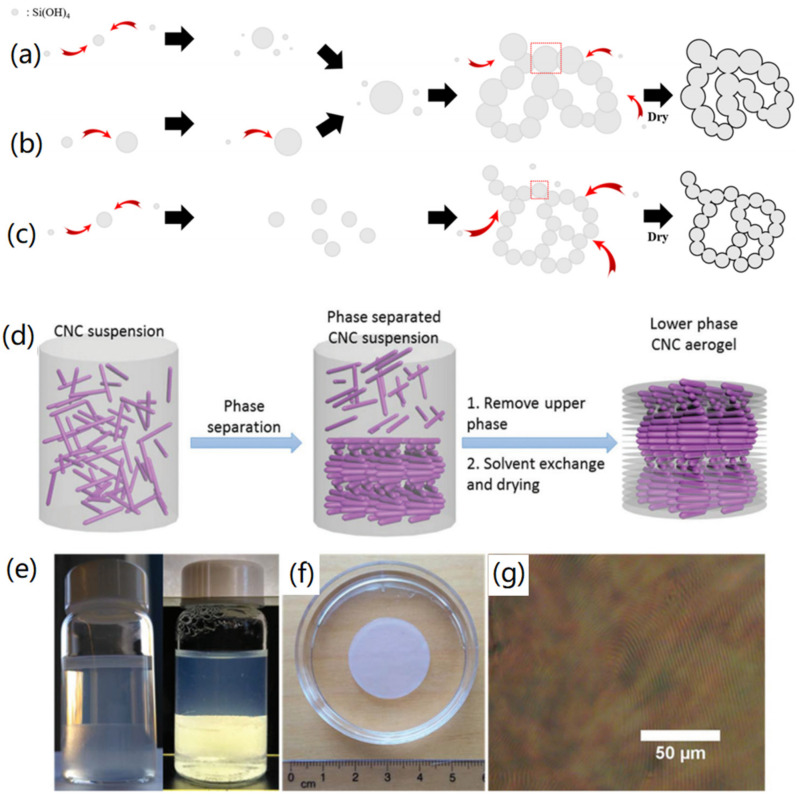
Structural tailoring of aerogels. (**a**–**c**) Porosity adjusting of aerogels via particle growth with different acidic feeding rate: (**a**) slow, (**b**) fast, and (**c**) optimized. Reprinted with permission from Ref. [32], Copyright 2019, MDPI. (**d**–**g**) Periodicity adjusting of cellulose nanocrystal (CNC) aerogels: (**d**) synthesis method, (**e**) CNC suspension without and with crossed polarizers, (**f**) CNC aerogels with cylindrical shape, and (**g**) polarized optical microscopy image of aerogels. Reprinted with permission from Ref. [34], Copyright 2018, Royal Society of Chemistry.

Different from the above-mentioned solvent exchange method, Ban et al. reported a method to prepare aerogels with different structures using silica to adjust the acidity and feeding rate, in order to change the porosity of the aerogels [32]. Their results showed that the colloidal gels at slow and fast feed rates had lower specific surface area, due to non-uniform particle size (Figure 2a,b), while, growing each cluster to form particles with the same particle size could effectively improve the porosity and specific surface area of the overall aerogels (Figure 2c). In another study, a cellulose aerogel with a hierarchical macroporous structure was designed by using the oil droplets in cellulose-dissolved molten salt hydrate as a structural template [33]. The hydrogel was dried by different drying methods, and it was found that the aerogels obtained by the supercritical drying process exhibited minimal shrinkage, with a finely distributed mesoporous structure and a diverse pore size distribution. The cellulose aerogels obtained by the freeze-drying method had macropores and hierarchical porous structure with different structural morphologies. However, the aerogels produced by ambient drying revealed large volume shrinkage, reduced overall porosity, and more aggregation. Therefore, it can be concluded that the drying methods are crucial for the structure of aerogels. Usually, supercritical drying exhibits better performances for the production of aerogels than both freeze-drying and ambient drying, although it needs more complex operation and has higher costs.

In addition to changing the solvent of the aerogel preparation process, it is possible to introduce some materials as a template during the aerogel assembly process to regulate the structure of aerogels [35]. In a previous review, Antonietti et al. summarized the process of synthesizing CAs and compared the structural characteristics of aerogels produced by different techniques, for example, those synthesized using silica as a hard template [36]. CAs generally have higher structural density, while soft template extends the hard templating process in terms of regularity, size, and morphological control of aerogels. In a typical study, Xu and co-workers reported the preparation of chiral CNC aerogels by taking advantage of the feature that CNCs can form a left-handed nematic liquid crystal phase via CO_2_ phase separation and supercritical fluid extraction [34], as shown in Figure 2d. Based on the prepared CNC aerogels, they further incorporated silica to form silica/CNCs composite aerogels. After calcination, CAs with a chiral nematic liquid crystal phase and periodic structure were produced. As shown in Figure 2e, a clear phase separation could be observed after 2–4 days for the CNC water suspension sealed in the vial. The solvent exchange process with ethanol was used for the gelation to obtain a cellulosic alcohol gel with a certain liquid crystal order (Figure 2f). It can be seen from Figure 2g that the CNC had birefringence and fingerprint texture features of chiral nematic order. The composite aerogel obtained by doping silica and CNC could perfectly retain the chiral nematic structure of CNC. After the CNC was removed by calcination, the periodic characteristics still existed, and the periodic structure could also be changed by changing the ratio between the two substances.

Besides the above methods, CMBAs can also be fabricated by the 3D printing technique. For instance, the 3D printing technique has been utilized for the fabrication of functional CAs [37,38] and GAs [39,40] with adjustable porosity and periodicity for various applications. As a facile 3D construction technique, 3D printing reveals high potential for regulating the structure and functions of CMBAs.

It should be noted that it is hard to synthesize pure CMBAs, as usually two or more material components are needed for the cross-linking and the formation of hydrogels, which could be transferred into aerogels by drying and/or subsequent high-temperature thermal carbonization. Therefore, CMBAs are usually as-prepared materials and the purity of CMBAs is based on the content of carbon in the final aerogels.

## 3. Synthesis of Carbon Material-Based Aerogels

In Section 2, we introduced the synthesis methodologies and structural regulation of various aerogels. In most of the cases, CMBAs can be synthesized through the formation of hydrogels with chemical and physical cross-linking, and subsequent drying and thermal reduction processes, based on the type of carbon resources used. In this section, we demonstrate the fabrication of functional CMBAs by using various carbon material precursors, including polymer-derived carbon, CNTs, CNFs, graphene, carbides, fullerenes, and carbon dots.

### 3.1. Polymer-Derived Carbon Aerogels

**Figure 3:**
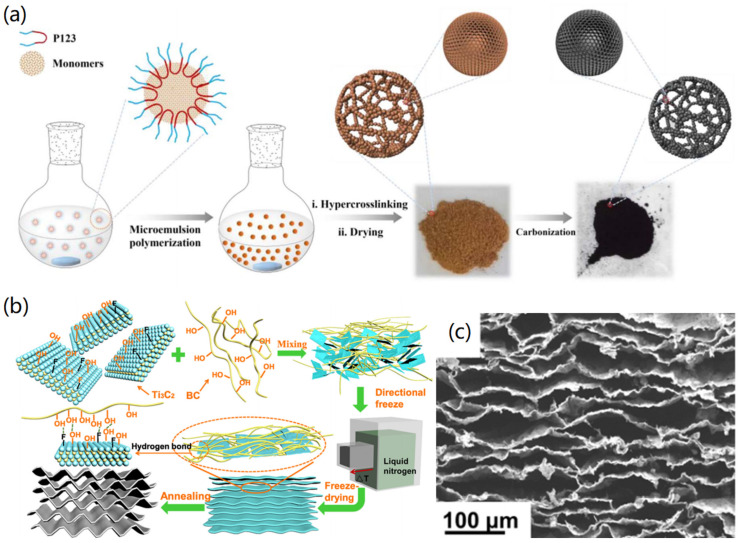
Fabrication of carbon aerogels: (**a**) Powdery carbon aerogels. Reprinted with permission from Ref. [41], Copyright 2017, Elsevier Ltd. (**b**,**c**) 2D titanium carbide and bacterial cellulose-derived carbon aerogels. (**b**) fabrication process and (**c**) SEM image of the carbon aerogels. Reprinted with permission from Ref. [43], Copyright 2019, American Chemical Society.

CAs and their precursor polymer aerogels have a unique 3D interconnected porous network structure, which can minimize the resistance of mass transport and are an important class of porous materials. In order to produce porous materials with high-performance, Xu et al. proposed a facile method to develop a new class of powdered carbon aerogels (PCAs) with high specific surface area [41], and the synthesis process is shown in Figure 3a. The polymerization of Pluronic 123 (P123)-stabilized microemulsion promotes the formation of poly(styrene-divinylbenzene) (PSDVB) nanoparticles, which are cross-linked via the Friedel-Crafts reaction to form the precursor powder polymer aerogel (PPA). After high-temperature carbonization for 10 h, powdery CA (PCA) was successfully obtained. The created PCA had a specific surface area as high as 2052 m^2^/g. The obtained SEM images showed that there was no obvious difference in the nanomorphology of PPA before the carbonization and PCA after the carbonization, indicating that the 3D interconnected nanonetwork structure had good nanostructure inheritance.

Since the nanoscale network structure can provide a very high surface area for the resulting CAs, Dang et al. directly dissolved cellulose into a mixture of NaOH (7 wt%), urea (12 wt%), and lanthanum (5 wt%), followed by gelation and carbonization, and successfully synthesized N, S dual-doped hierarchical porous CAs [42]. It was found that the formed CAs had an amorphous slot hole-like porous structure, which was clearly observed by both field emission scanning electron microscope (FE-SEM) and transmission electron microscope (TEM) characterizations. As the formed CAs revealed highly specific surface area and hierarchically porous networks, they could be potentially used for the adsorption of various molecules and ions for environmental and energy science applications.

As a new generation of functional materials, the electrochemical inertness of CA gives it great potential in the field of electrochemistry. Chen et al. prepared a compressible and elastic CA (CECA) with high flexibility and elasticity using biomass template [43]. The preparation process is shown in Figure 3b, where the MXene (Ti_3_C_2_) nanosheets were first connected to a continuous nanosheet structure by using bacterial cellulose (BC) nanofibers as a nano-binder, and lightweight CECA was prepared by directional freeze-drying and carbonization. It was observed from the SEM image (Figure 3c) that the formed CECA material showed parallel and continuous flake structure after annealing. It is clear that in this hybrid aerogel, BC promoted the continuous assembly of Ti_3_C_2_ nanosheets, while Ti_3_C_2_ nanosheets induced parallel arrangement of BC layers, due to their 2D sheet feature. These properties made the CECA-based sensor a flexible wearable device that couldd monitor subtle and large biological signals from the human body.

In addition to the excellent properties of light mass, high porosity, low density, and high specific surface area of general aerogels, CMBAs also have excellent conductivity, relatively good mechanical properties, and good acid and alkali resistance. The advantages of carbon materials mean that they are widely used in thermal, acoustic, electrical, catalytic, hydrogen storage, and nuclear physics research fields. For example, Han et al. synthesized lightweight, hydrophobic, and porous CAs from waste newspaper as the only raw material [44]. The synthesized porous CAs had a low density of 18.5 mg/cm^3^ and a water contact angle of 132°. In addition, the adsorption capacity of CAs towards organic solvents and oils was 29–51 times their own weight, and, therefore, could be used as an economical, efficient, and safe absorbent for both environment and marine protection. Wang et al. prepared CAs through the coating of melamine foam (MF) with TEMPO-oxidized cellulose nanofibers (TOCN) after the pyrolysis of biomass materials at high temperature [45]. The formed CAs exhibited low density (11.23 mg/cm^3^), high nitrogen content (6.35%), high compressibility (60%), and high conductivity (0.378 S/cm). In another case, Chen and co-workers prepared sustainable CAs with nanofibrillated cellulose (NFC) as the precursors [46], which revealed significant adsorption capacity for various oils and organic solvents, due to their low density (about 7.8 mg/cm^3^), high porosity, high elasticity, good hydrophobicity, and good lipophilicity.

### 3.2. CNT-Based Aerogels

**Figure 4:**
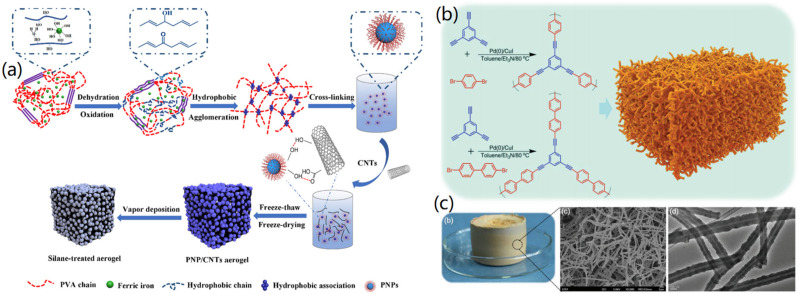
Fabrication of CNT-based aerogels: (**a**) PVA-based nanoparticles (PNP)/CNTs aerogels. Reprinted with permission from Ref. [47], Copyright 2020, Elsevier Ltd., (**b**,**c**) Hollow CNT aerogels: (**b**) synthesis process and (**c**) photograph and microscopy characterizations. Reprinted with permission from Ref. [6], Copyright 2019, Wiley VCH.

The unique inherent properties of CNTs make them widely used as enhancement components to various composite aerogels, and, meanwhile, their nanoscale structure is perfect for achieving CMBAs with high porosity and interconnected nano-networks. Zhang and co-workers developed a novel synthesis strategy of CNT-based aerogels by combining PVA-based nanoparticles (PNPs) and CNTs [47]. The synthesis process is shown in Figure 4a, where, in the presence of Fe^3+^ ions, CNTs were introduced into the PVA solution for hydrothermal treatment, and the resulting PNP/CNTs solution was obtained, which could be further treated to PNP/CNTs aerogels through a freeze-drying process. The materials characterizations indicated that PNPs presented a dandelion-like structure, with cross-linked molecular chains as the core and uncross-linked PVA chains as the shell on the PNP surface. In addition, it was found that there were many CNTs on the surface of PNP/CNTs aerogels, which tangled with PNPs to form a composite structure. The unique properties of the synthesized PNP/CNT aerogels, such as good hydrophobicity, high porosity, and interconnected porous structure, revealed great potential for adsorption of organic solvents in environmental science.

Based on the interactions between BC and multi-walled carbon nanotubes (MWCNTs), Hosseini and co-workers synthesized a flexible lightweight conductive BC/MWCNT composite aerogel via supercritical CO_2_ drying [48]. Their study indicated that after MWCNTs were introduced into the medium, the average pore size and volumetric shrinkage of BC/MWCNTs nanocomposite aerogels decreased to 8.6 nm and 2%, respectively, and the specific surface area and bulk density soared to 235 m^2^/g and 0.024 g/cm^3^, respectively. In another study, Dong et al. prepared CMCs (carboxymethyl cellulose)-modified CNT aerogels through the freeze-drying method [49]. The formed CMC/CNT hybrid aerogels had low bulk density, high mechanical strength, and good processability. The simple preparation method, controllable hierarchical microstructure and versatility of this CNT aerogel provides a new idea for the production and utilization of aerogel materials.

In addition, various nanoparticles can also be added into CNT-based aerogels for the fabrication of function-reinforced hybrid CMBAs [50,51]. For instance, Jeong et al. converted B_2_O_3_ nanoparticles into hexagonal boron nitride (h-BN) in an aerogel of single-walled carbon nanotubes (SWCNTs) by pyrolysis at 1000 °C [51]. The fabrication included the h-BN coating process in the synthesis of flexible aerogels, creating functional hybrid CMBAs with ultra-compressive ability, high porosity, and excellent elastic recovery properties. This technique is very advantageous for the preparation of SWCNT-based porous composites of ceramic materials, including fragile SWCNT films and aerogels.

In order to obtain CNT-based aerogels with hierarchical nanoporous structure, Mu et al. synthesized monolithic CNT-based aerogels by the carbonization of self-assembled polymer nanotubes [6]. The synthesis mechanism of the conjugated microporous polymer aerogel (CMPA) is shown in Figure 4b, in which Pd(0)/CuI was used as the catalyst, and triethylamine and toluene served as co-solvents. It was found that the formed CMPA revealed a hollow nanotube structure with a diameter of approximately 100 to 250 nm (Figure 4c). The created CNT-based aerogels had rich porosity (92%), high specific surface area (826 m^2^/g), low density (57 mg/cm^3^), wide light absorption (99%), low thermal conductivity (0.192 W m^−1^ K^−1^), extensive light absorption (99%), and a super-hydrophilic open channel structure.

CNT-based aerogels can also be fabricated through the chemical vapor deposition (CVD) method. For instance, Khoshnevis et al. synthesized lightweight, hydrophobic, and porous CNT aerogels using the floating catalyst–chemical vapor deposition (FC-CVD) method with toluene as the carbon source [52]. The created CNT aerogels revealed high adsorption capacity, high adsorption rate and good reusability for oil adsorption, showing high potential in environmental science. In a similar study, Mikhalchan and co-workers utilized the FC-CVD method to prepare highly porous and ultralight CNT aerogels [53], which exhibited low density, high porosity, and high conductivity. These CNT-based aerogels have been widely used for liquid adsorption and energy storage applications.

### 3.3. CNF-Based Aerogels

**Figure 5:**
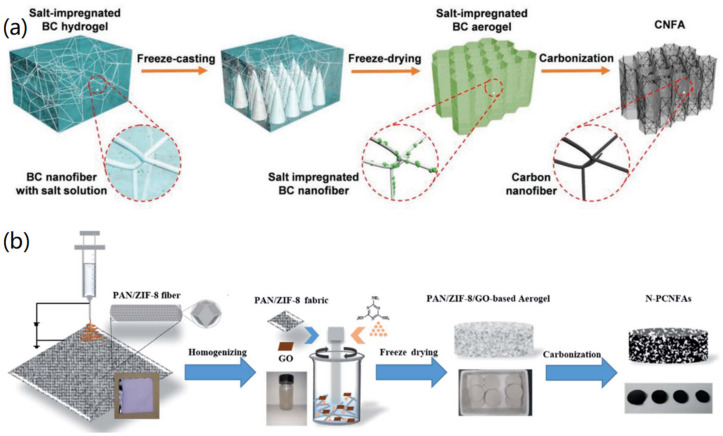
Fabrication of CNF-based aerogels: (**a**) BC nanofiber-based fabrication of CNF aerogels. Reprinted with permission from Ref. [56], Copyright 2020, Wiley VCH. (**b**) Electrospinning, freeze drying, and carbonization-based formation of CNF aerogels. Reprinted with permission from Ref. [59], Copyright 2020, Royal Society of Chemistry.

CNFs can be used as good precursor carbon materials for the fabrication of functional CMBAs. CNFs are synthesized through the carbonization of biomass (such as cellulose, chitosan, chitin, and others) nanofibers and electrospun polymer nanofibers [54,55]. BC nanofibers have a large length-to-diameter ratio compared to other types of nanocellulose. Li et al. reported the fabrication of CNF-based aerogels (CNFAs) through the formation of BC nanofiber aerogels and subsequent carbonization [56], as shown in Figure 5a. It is a facile and effective method for the fabrication of CNFAs. The obtained results indicated that, after high-temperature carbonization, the hierarchical honeycomb cellular structure and the nanofiber junction structure was perfectly inherited in the CNFA.

Through a similar method, Ding and co-workers prepared CNF-based aerogels through the gelation and carbonization of chitin nanofiber aerogels [57]. The formed CNF aerogels had a homogeneous nanofiber structure and N-rich composition, and, therefore, could be used as an effective adsorbent of dyes, providing a new and powerful way to build promising energy storage and environmental remediation materials from chitin waste. In another study, Li et al. developed a facile method to prepare mechanically stable ultra-thin CNF aerogels by thermal decomposition of wood-based NFC aerogels [58]. The obtained results showed that highly homogeneous CNFs retained the morphology of the nanofibers well during pyrolysis and converted into interconnected ultrafine CNFs. In addition, the wood-derived CNF aerogels revealed a stacked nanosheet structure, excellent electrical conductivity, and exhibited high compressive strength.

To improve the function and properties of CNF-based aerogels, various 2D materials and nanoparticles have been added into the aerogel systems for the fabrication of hybrid CMBAs. For instance, Yang et al. prepared compressible elastic N-doped porous carbon nanofiber aerogels (N-PCNFA), and the synthetic route is shown in Figure 5b [59]. Using ZIF-8 nanoparticles as pore templates, PCNF was synthesized with electrospun PAN nanofibers, and then the mixture was freeze-dried after homogenization to obtain a precursor aerogel composed of melamine (melamine), PAN/ZIF-8 nanofibers, and GO. After carbonization at 1000 °C, ultra-low density elastic N-PCNFA was produced. The formed N-PCNFA was found to retain its cellular framework, and the structure of the rGO-wrapped cross-PCNF and coiled PCNF strengthened the 3D fiber backbone to avoid its collapse. The abundant pore structure allowed N-PCNFA to obtain a large specific surface area and a robust structure was constructed by introducing mechanically reinforced structures, which promoted the adsorption and desorption of ions and revealed potential application in electrochemical catalysis and energy storage.

By treating natural BC containing molybdenates through a simple solid-state reaction, Liu et al. synthesized MoC nanoparticle-embedded CNF aerogels (MoC@CNFAs) [60]. The FESEM results showed that MoC@CNFAs were 3D network structures composed of many nanofibers, which not only had many nodes, but also expanded the accessible surface area and shortened the ion transport path. Strong mechanical strength was also evident, forming an aerogel structure that allowed water to pass through. The TEM analysis revealed that there were ultra-small MoC nanoparticles with a size of about 5 nm embedded in thin CNFs. The synthesized CMBAs exhibited excellent desalination performance with fast rates and high capacity.

### 3.4. Graphene-Based Aerogels

As an excellent 2D carbon material, graphene is commonly used to synthesize 3D Gas, based on its stable mechanical structure and excellent electrical and heat transfer properties. Graphene-based CMBAs exhibit other excellent properties on the basis of maintaining the original excellent properties of graphene, such as having a large adjustable specific surface area and rich and adjustable porosity.

In 2009, Wang and Ellsworth, for the first time, reported the preparation of graphene aerogels through ultrasonic-induced gelation of GO and subsequent freeze-drying and thermal reduction of GO to graphene [61]. In another case, Compton and co-workers reported the synthesis of GO hydrogels via ultrasonication treatment on the precursor dispersions [62]. It was found that the gelation could be induced after 30 min, forming a relatively weak hydrogel. Extend ultrasonication for 120 min induced the formation of a hydrogel with a highly stable structure. Therefore, ultrasonication treatment is important to regulate the structure and pore size of the formed hydrogels and aerogels, which can also affect the adsorption efficiency towards gases.

**Figure 6:**
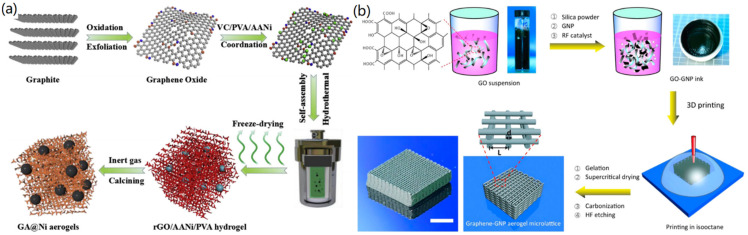
Fabrication of graphene-based aerogels: (**a**) GA@Ni hybrid aerogels. Reprinted with permission from Ref. [27], Copyright 2019, Elsevier Ltd. (**b**) 3D printing-created GAs with periodic macropores. Reprinted with permission from Ref. [63], Copyright 2016, American Chemical Society.

At present, GAs have been widely used for sensors, adsorbents, lightweight structural materials, catalysis, and other fields. For example, Xu et al. developed a class of 3D magnetic GAs with superior microwave absorption capacity through the combination of hydrothermal synthesis and in-situ pyrolysis [27]. As shown in Figure 6a, graphite was first oxidized by the hummers method to obtain the GO suspension, which was then mixed with vitamin C, PVA, and acetylaceton nickel (AANi) for the formation of rGO/AANi/PVA hydrogel after cooling. After carbonization, the rGO/AANi/PVA hydrogel was transferred into a magnetic 3D graphene aerogel (GA@Ni). Ascribing to the doping of ultra-fine Ni nanocrystals with a size of 8 nm, the created GA@Ni revealed excellent microwave absorption capabilities in practical applications.

As a raw material with good electrical conductivity, Zhu et al. used graphene to manufacture a 3D printed graphene composite aerogel (3D-GCA) for supercapacitor applications through 3D printing technology [63]. The preparation process is shown in Figure 6b, in which the GO precursor suspension, graphene nanosheets, silica filler, and catalyst were mixed to form a homogeneous, high-viscosity ink, and then the composite ink was printed with a 3D structure of GN-GNP. Finally, 3D-GCA was obtained by freeze-drying and high temperature carbonization. It can be seen that a thin wooden pile cubic lattice, consisting of multiple parallel cylindrical filament orthogonal layers, was constructed with a large specific surface area of 400–700 m^2^/g. In another study, Zhang et al. also used 3D printing technology to process the GO suspension into a GA with a 3D honeycomb-like porous structure on its surface [64]. In addition, Li et al. prepared an ultra-elastic high-strength GA coupled with GO suspension by using MF as the sacrificial skeleton [65]. The SEM characterizations showed that GA retained an ordered porous structure with a pore size of tens of microns, which favored the excellent elasticity of the aerogel.

Xu et al. prepared a naturally dried graphene aerogel (NDGA) by borate-mediated hydrothermal reduction, using ordinary dialysis and pre-freezing techniques [66]. The formed NDGA had excellent properties, such as super reversible compressibility (99%), high conductivity (about 1.3 cm^−1^), and low thermal conductivity (0.018 W m^−1^ K^−1^). This study provides a new application path for the preparation and application of CMBAs in pollution prevention and electric nanodevices.

### 3.5. Carbide-Based Aerogels

Carbide materials have high hardness, excellent oxidation resistance, and good electrical and thermal conductivities, and can be used as precursors to prepare CMBAs. The prepared carbide aerogels contain a 3D network structure which can greatly reduce thermal conductivity and further improve the thermal insulation performance of the materials.

**Figure 7:**
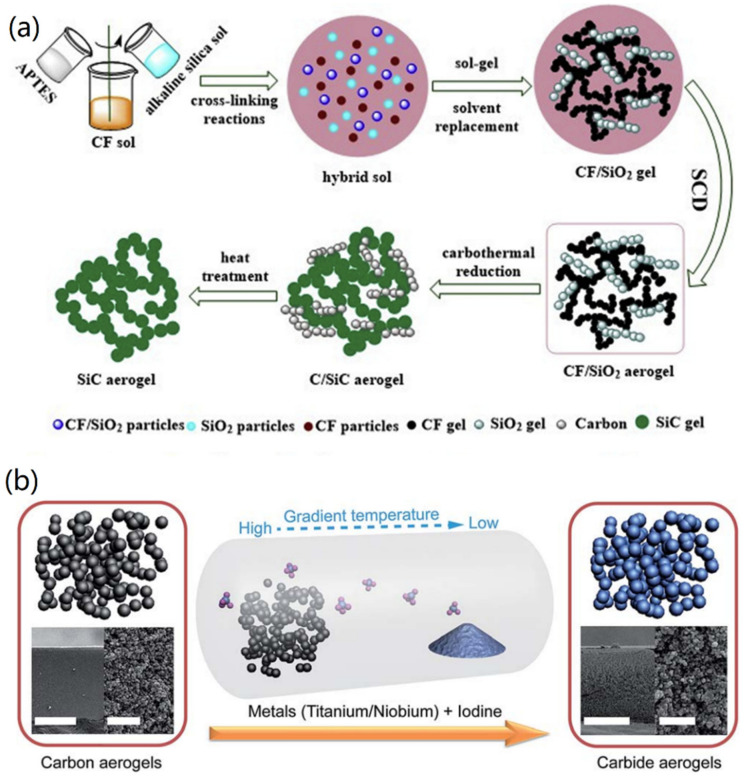
Fabrication of carbide-based aerogels: (**a**) SiC aerogels. Reprinted with permission from Ref. [67], Copyright 2019, Elsevier Ltd. (**b**) TiC and NbC aerogels. Reprinted with permission from Ref. [68], Copyright 2015, Royal Society of Chemistry.

An et al. converted a catechol formaldehyde/silicon (CF/SiO_2_) composite aerogel into an integral silicon carbide (SiC) aerogel through thermal reduction and calcination techniques [67]. As shown in Figure 7a, phthalone was dissolved in a mixture of formaldehyde and ethanol, and the mixed solution was obtained after magnetic stirring at room temperature for 0.5 h. Then, alkaline silica sol and triethoxysilane were added into the mixed solution to form the hydrogel via chemical cross-linking. The fabricated CF/SiO_2_ aerogel was then obtained by supercritical drying and carbonization at 800 °C for 3 h, followed by heating to 1500 °C for 5 h to form a C/SiC aerogel. The C/SiC aerogel was then transferred into a muffle furnace for 2 h under a gas stream at different temperatures to deplete the free C to obtain SiC aerogel. The formed SiC aerogels exhibited the same 3D structure as conventional aerogels, in which particles come together to form the structure of a porous network.

However, thermal reduction and calcination technologies are costly and time-consuming. Due to the various crystal growth trends of carbides at high temperatures, the morphological control of carbide aerogels is also difficult. To solve this problem, Chen and co-workers used a low-temperature pseudo-crystalline synthesis method for the synthesis of carbide-based aerogels [68]. In their study, CAs were converted directly into highly porous TiC aerogels without the need for a highly pure, protective atmosphere, such as Ar, as well as special heating systems. As shown in Figure 7b, the added gaseous metal iodides were adsorbed onto a porous 3D disordered frame of the pre-formed CAs to generate TiC or NbC aerogels, respectively. Through the SEM analysis, a porous morphology similar to that of the original CA template was maintained. The surface and pore size characterizations of the formed carbide aerogels were performed by nitrogen adsorption/desorption analysis. It was proved that these carbide aerogels exhibited higher specific surface area and lower bulk density than CAs. This proposed route can be used to prepare other types of carbide aerogels from Cas, via a relatively quick, economic, and mild strategy.

Besides, thermal reduction and carbonization have been utilized for the fabrication of other SiC composite aerogels [69,70,71]. These studies not only provided possible avenues for the preparation of carbide aerogels, but also presented broader prospects for future applications of carbide aerogel materials in thermal insulation materials, telecommunications equipment, and other fields.

### 3.6. Other Carbon-Based Aerogels

In addition to the above introduced carbon materials for the fabrication of various CMBAs, 0D carbon materials, such as fullerenes and carbon dots, can also be used to fabricate CMBAs. However, there are only limited references on the fabrication of fullerene and carbon dot aerogels, in which fullerene and carbon dots were used as the doping components for the fabrication of hybrid aerogels. For instance, Shen et al. reported the doping of silica aerogels with fullerenes (C_60_ and C_70_) to synthesize hybrid aerogels with enhanced photoluminescence [72]. It was found that the luminescent intensity of the formed aerogels changed with the adjustment of the fullerene content in the aerogels. Dolai and co-workers reported the synthesis of carbon dot/polymer hybrid aerogels via an in-situ synthesis method, and the formed hybrid aerogels could be used for the fabrication of fluorescent sensors for detecting VOCs [73]. In another study, they synthesized carbon dot aerogels through in-situ polymerization and carbonization of 2-thenoyltrifluoroacetone (TTA), and found that the formed carbon dot aerogels enabled the effective detection of UO_2_^2+^ ions [74].

To make the above clearer, we provide a table (Table 1) summarizing the materials, structures, properties, and potential applications of various CMBAs.

## 4. CMBAs for Gas Adsorption

CMBAs have shown wide applications in the fields of solution adsorption, energy storage, electrochemical catalysis, sensors/biosensors, drug delivery, tissue engineering, and many others. In this section, we focus on the gas adsorption of CMBAs, including the adsorption of CO_2_, CO, H_2_S, Hg vapor, H_2_, and organic gases.

### 4.1. Adsorption of CO_2_ and CO

Polymer aerogels can be carbonized to CAs, which exhibit great potential for the adsorption of CO_2_. For instance, Robertson et al. synthesized a highly microporous CA by the gelation and carbonization of resorcinol-formaldehyde (RF) resin by means of the supercritical drying method, and further produced activated CA (ACA) by KOH activation of CA [75]. The created ACA had high microporosity, with an increase in surface area of 80% to 290% and an increase in total pore volume of 46–200% after activation at a range of temperatures. In the CO_2_ adsorption experiments, the CAC showed a high level of adsorption capacity of 2.7–3.0 mol/g, ascribed to sufficient pores in the range of less than <15 Å in the structure of ACA. In other cases, polymer aerogels synthesized by tris(4-isocyanatophenyl) methane/pyromellitic [76] acid and pyrrole/formaldehyde [77] were carbonized to CAs for the adsorption of CO_2_. It was found that the formed CAs had high porosity and large BET surface area after the two-step process of carbonization and etching. In addition, the CAs prepared by polymer aerogels revealed high stability, good selectivity, and excellent sustainability.

The calcination temperature can affect the porous structure of CAs, which has a crucial effect on the adsorption ability of CAs. For instance, Liu et al. explored the effect of the calcination temperature on the CO_2_ adsorption performance of CAs produced with the microemulsion template method [78]. They found that with increase of calcination temperature, the surface area and pore volume range expanded in the range of 600–800 °C. However, the surface area and pore volume decreased after temperatures were higher than 800 °C, which might have been due to the collapse of the aerogel carbon layer due to the excessively high temperature. In optimal conditions, the synthesized CA revealed the largest adsorption capacity of 93.98 cm^3^/g and the lowest desorption temperature (55 °C). This study showed that the distribution of pore size and the size of the surface area have important effects on the CO_2_ adsorption capacity of CAs.

Biomass (such as cellulose, chitosan, and lignin)-based CAs have also been widely used for the adsorption of CO_2_ in recent years [79,80,81]. Hu et al. first prepared cellulose aerogels and then annealed the cellulose aerogels at high temperature in an NH_3_ atmosphere to prepare N-doped CAs with an N content of 4.62 wt%, rich hierarchical porous structure, and excellent CO_2_ adsorption performance [81]. The obtained results indicated that the N-doped CA (CA-800), prepared by annealing at 800 °C, had a larger surface area than the non-N-doped CA (N-free CA-800) at the same temperature. It is clear the high temperature annealing in NH_3_ atmosphere forms more micropores and mesoporous structures, which increases the large surface area of the aerogels. In the CO_2_ adsorption experiment, the large amount of microporous structure and N-containing groups of N-doped CA-800 promoted its CO_2_ adsorption capacity to reach 4.99 mmol/g, which was much higher than that of N-free CA-800 of 3.56 mmol/g. In another case, Alhwaige and co-workers innovatively used bio-based chitosan and polybenzoxazine (PBZ) as reactive precursors, and montmorillonite (MMT) as a composite reinforcement material to prepare MMT-CTS-PBZ nanocomposite CAs with high CO_2_ adsorption performance [82]. The addition of MMT greatly increased the pore volume and surface area of the CAs, which was beneficial to the adsorption of CO_2_. When the ratio of MMT, CTS, and PBZ was 1:1:1, the porosity of MMT-CA reached a maximum of 0.319 cm^3^/g, and the absorption of CO_2_ reached a maximum of 5.72 mmol/g. In the adsorption and desorption cycle experiments, the adsorption capacity decreased by only 2% after 6 cycles, indicating high stability of the synthesized CAs towards CO_2_ adsorption.

**Figure 8:**
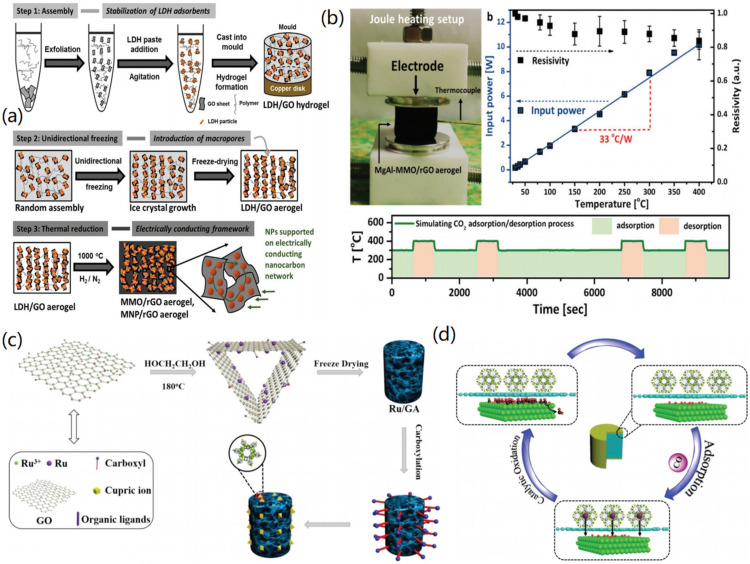
Carbon material-based aerogels for the adsorption of CO_2_ and CO. (**a**,**b**) MgAl-MMO/rGO hybrid aerogels for the adsorption of CO_2_: (**a**) synthesis process, and (**b**) adsorption and desorption of CO_2_. Reprinted with permission from Ref. [83], Copyright 2020, Wiley VCH. (**c**,**d**) 3D Ru/GA-MOF aerogel for the adsorption and catalysis of CO: (**c**) fabrication of Ru/GA-HK aerogels, and (**d**) reaction mechanism for CO removal. Reprinted with permission from Ref. [85], Copyright 2018, Wiley VCH.

2D materials can be utilized for the synthesis of CMBAs for the adsorption of CO_2_ and CO gases, in which graphene materials have been widely applied. For instance, Xia et al. designed GO-based aerogels by combining layered double hydroxide (LDH)-derived nanoparticles and MgAl nanoparticles together [83]. As shown in Figure 8a, 3D LDH/GO hydrogels were first formed through the self-assembly process of LDH particles and GO nanosheets. Ice-templated macroporosity was then induced by unidirectional freezing, and the columnar LDH/GO aerogels were obtained after freeze-drying. Finally, high-temperature annealing was applied in the reducing atmosphere to form the MgAl-LDH/rGO aerogels. The DBT adsorption model of the MgAl-LDH/rGO mixed aerogel was evaluated in an organic sulfur adsorption experiment, and it was found that the maximum DBT absorption capacity of the MgAl-LDH/rGO aerogel was twice that of the MgAl-LDH powder. On the other hand, the composite CMBA exhibited excellent thermal stability and electrical conductivity, due to the support of the rGO aerogel framework. The resistivity of the MgAl-LDH/rGO aerogel underwent only minor changes at higher temperatures (Figure 8b), and these changes were reversible. It could be observed that the MgAl-LDH/rGO aerogel showed an excellent CO_2_ adsorption effect under high temperature and high pressure, reaching 2.36 mmol/g, and the adsorption capacity was increased by 160% compared with the MgAl-LDH powder. More than 95% of the maximum adsorption capacity was maintained even after 5 cycles. The above experimental results demonstrate the excellent stability and excellent adsorption effect of designed complex aerogels. In another study, Liu et al. fabricated 3D glucose-graphene-based aerogels (G/GAs) with excellent adsorption effect by the hydrothermal method and CO_2_ activation, using glucose as a cross-linking agent [84]. Using glucose to replace the traditional cross-linking agent provides good degradability and biocompatibility, which improves the environmental friendliness. The as-prepared G/GAs had high mechanical strength and a uniform hierarchical pore structure with a 3 nm-dominated pore size distribution and a specific surface area as high as 763 m^2^/g, resulting in a CO_2_ absorption capacity as high as 76.5 mg/g. In addition, the synthesized graphene aerogels had a good absorption effect on CH_4_ and H_2_.

2D MOF materials are excellent precursors for the fabrication of CMBAs for CO_2_ adsorption. For instance, Ren et al. prepared a mild room-temperature synthesis of MOF (CuBTC)/GA hybrid aerogels using ionic liquid (IL) as the additive [86]. The surface area of CuBTC/GA-IL was slightly increased compared with the CuBTC/GA aerogel, which was beneficial to the adsorption of CO_2_. The CO_2_ absorption rate of CuBTC/GA-IL was as low as 3.71 mmol/L, and the CuBTC/GA-IL exhibited lower mass transfer resistance and more stable vicious cycle performance in the dynamic adsorption experiment. This method of incorporating MOF materials into microscopic pores of GA using IL additives provides an effective design idea for future applications of MOFs and CMBAs. To improve the adsorption of CMBAs towards CO, Qu and co-workers designed a hydrothermal method to modify the HKUST-1 (HK)-containing MOF and Ru onto the surface of GA to prepare 3D Ru/GA-HK [85]. As shown in Figure 8c, the Ru/GA was synthesized by the hydrothermal method, and then the Ru/GA was modified with APTES and succinic anhydride to conjugate the HK-MOF. Finally, the Ru/GA-HK was prepared by freeze-drying, carbonization, and step-by-step assembly. The obtained Ru/GA-HK exhibited high adsorption of CO and a conversion of 19.8% at 30 °C, which was about 48.4% higher than that of Ru/GA. In addition, the conversion rate reached 100% at 150 °C, and the catalytic activity remained stable for 48 h. As shown in Figure 8d, the 3D porous structure of the Ru/GA-HK was more conducive to the diffusion and contact of CO molecules during the catalytic process, so that the concentration of CO inside the aerogel increased and reacted with the co-adsorbed O to generate CO_2_ and release it from the Ru surface. The designed Ru/GA-HK showed excellent application prospects for CO adsorption and catalysis.

Besides the above-mentioned materials, CNTs can also be used in combination with polymers for the fabrication of ultralight CMBAs with good mechanical properties. For instance, Gromovsen et al., for the first time, designed ultralight aerogels with good mechanical properties using oxidized-CNTs and polyvinyl alcohol (PVA) [87]. The fabricated CNT/PVA aerogels had very high internal volumes (35–70 mL/g) and could be used as the amine-impregnated support for CO_2_ adsorption. The CNT/PVA aerogel kept good pore structure when the amino-loading content was 85%. For practical applications, the amine-impregnated CNT/PVA aerogels exhibited excellent performance in simulated fossil fuel combustion flue gas, with the highest adsorption capacity reaching 3.3 ± 0.3 mmol/g and an amine utilization rate of 35%. This material has excellent capture performance at atmospheric pressure or at low CO_2_ concentrations.

### 4.2. Adsorption of H_2_S and Hg Gases

H_2_S is a highly irritating toxic gas, which is mainly derived from wastewater, garbage disposal, natural gas, or the oil production and refining processes. In many synthetic refining processes, the release of H_2_S occurs, and brings serious corrosion to the entire process facilities. H_2_S released into the air is converted into sulfur oxides of acid rain and PM2.5, the main precursors, which not only cause serious air pollution, but also seriously damage the health of human beings. Therefore, the adsorption and removal of H_2_S to protect the atmospheric environment is of vital importance to pollution control and safety protection of current industrial processes.

In order to remove H_2_S from the air, Chen et al. reported the synthesis of a N-doped porous carbon (NPC) prepared by hydrothermal carbonization and activation of waste polyurethane (PU) foam, where the hydrothermal solution was cross-linked with glutaraldehyde through hydrolysis and decarboxylation during the pretreatment stage [88]. After the polymerization, mixing with K_2_CO_3_ aqueous solution was conducted, and, subsequently, carbonization. Functional porous CAs were produced, which exhibited a dense interconnected porous structure. When H_2_S passes through the CA, it oxidizes and induces the deposition of sulfur and sulfates. Compared to pristine porous carbon, the created CAs had higher desulfurization performance, and the saturated sulfur capacity could reach 205.06 mg/g.

**Figure 9:**
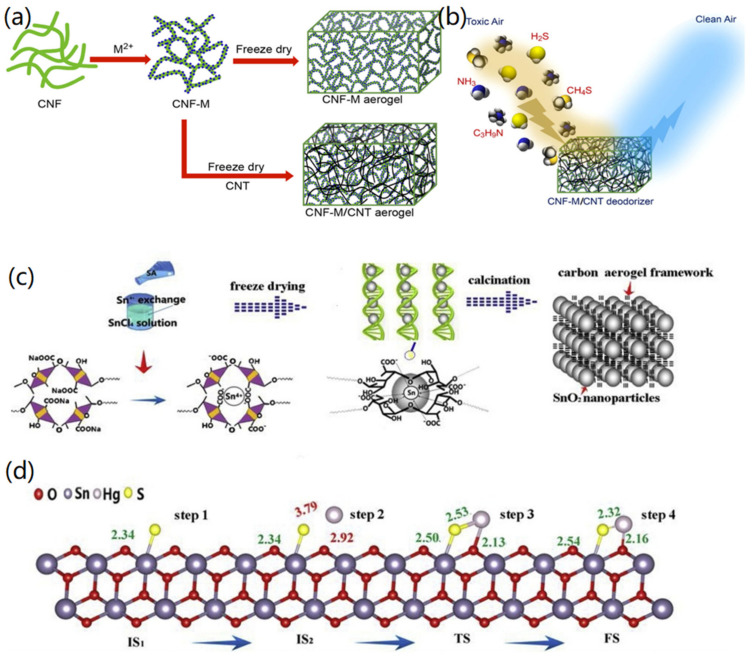
Carbon-based aerogels for the adsorption of H_2_S. (**a**,**b**) CNT/CNF-M^2+^ hybrid aerogels for the adsorption of H_2_S and other gases: (**a**) synthesis process and (**b**) adsorption and removal mechanism. Reprinted with permission from Ref. [89], Copyright 2021, Elsevier Ltd. (**c**,**d**) 3D SnO_2_/CA for the adsorption of H_2_S: (**c**) synthesis process, and (**d**) reaction mechanism. Reprinted with permission from Ref. [90], Copyright 2019, Elsevier Ltd.

Biomass-based CMBAs have been widely used for the adsorption of H_2_S gas. For instance, Kiliyankil et al. developed a facile method to synthesize CMBAs using TEMPO-oxidized cellulose nanofibers (CNFs), CNTs, and transition metallic ions (M) [89]. As shown in Figure 9a, both CNF-M and CNF-M/CNT aerogels could be synthesized by metallic ion-induced cross-linking and subsequent freeze-drying. Through the calculation, the total pore capacity of the CNF-M/CNT hybrid aerogel was calculated to be 0.49 cm^3^/g, and the porosity reached 99.17%, conducive to the capture of toxic gases in the air. In the ammonia adsorption experiment of 150 ppm, the clearance rate of CNF-Cu/CNT within 10 min reached 95%, and the removal efficiency within 30 min reached 99.3. For the adsorption effect of methanethiol, the CNF-Cu/CNT aerogel could completely adsorb 100 ppm of methanethiol within 30 min. In addition, the removal efficiency towards H_2_S reached 80% adsorption within 10 min and complete removal within 30 min. The removal efficiency was remarkable even at higher concentrations of harmful gases. The reaction mechanism is presented in Figure 9b. When harmful gases passed through the aerogel, gas molecules reacted with the metallic ions in the CNF-M/CNT aerogel to promote the adsorption of gas molecules. For example, H_2_S molecules could react with Cu^2+^ to generate a solid complex fixation. In addition, the presence of CNTs also play a crucial role in the adsorption of H_2_S molecules through physical adsorption.

Zhang et al. synthesized SnO_2_/CA using sodium alginate (SA) as a template [90]. As indicated in Figure 9c, Sn^4+^ replaced the Na^+^ in SA to form an Sn-alginate hydrogel, which was then turned into a 3D porous CA by freeze-drying and carbonization at high temperature. The created SnO_2_/CA had a surface area of 142.25 m^2^/g and a pore volume of 0.20 mL/g, with a clear pore hierarchy, conducive to rapid adsorption of high flow rate gases. As shown in Figure 9d, the theoretical calculation indicated that the adsorption mechanisms of SnO_2_/CA on H_2_S and Hg vapor gases, for which the SnO_2_ surface-adsorbed H_2_S has a low energy barrier, resulted in easy generation of HgS. First of all, SnO_2_ can adsorb H_2_S and produce the active substance SnS_2_ and element S, and the adsorbed sulfur reacts with gaseous Hg to generate HgS. At 30–120 °C, the synthesized SnO_2_/CA revealed complete adsorption capacity for H_2_S and Hg gases, and the removal efficiency of H_2_S reached 95% in a 10 h test, and the adsorption capacity was 392.23 mg/g. In addition, after 5 cycle regeneration experiments, the SnO_2_/CA still exhibited the adsorption capacities of 156.30 μg/g and 343.61 mg/g towards Hg and H_2_S gases, respectively, indicating the adsorption performance of the designed functional CMBAs.

In addition, the addition of metallic nanoparticles into CMBAs could enhance the adsorption performance of aerogels to H_2_S. For instance, Tian et al. developed a novel synthesis method to prepare CAs with efficient desulfurization performance by fixing Fe NPs onto the CA skeleton structure [91]. The obtained experimental results showed that the Fe NPs-fixed CA had good anti-H_2_ ability and the best desulfurization effect on hot H_2_S gas at 600 °C, reaching an adsorption capacity of 12.54 g/100g, which was the frontrunner among various hot gas desulfurization adsorption materials. In another study, Zhang and co-workers developed a AgNP-doped CA sorbent using fulvic acid as a template for the synthesis of uniformly loaded AgNPs [92]. The designed CA could be effectively used for the adsorption of H_2_S and Hg gases. In the 10h test, the created CMBAs exhibited an adsorption capacity of 1.36 mg/g and adsorption efficiency of above 99% for Hg gas, ascribed to the improved capture and adsorption of small AgNPs to Hg gas.

### 4.3. Adsorption of H_2_

CMBAs can be utilized for the adsorption of elemental gases, such as H_2_. The adsorption and storage of H_2_ play an important role in replacing conventional coal and petroleum energies, and promotes the preparation of new energy materials with high efficiency, low cost, and neglectable environmental pollution. CAs are attractive materials for the adsorption and storage of H_2_ due to their porous structure, ultralight mass, high surface area, and abundant adsorption sites [93].

**Figure 10:**
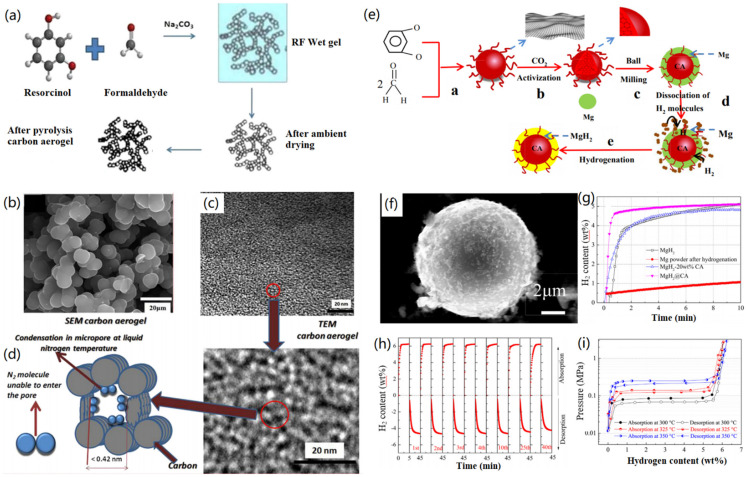
CMBAs for the adsorption of H_2_. (**a**–**d**) Pt-modified CAs for the adsorption of H_2_: (**a**) synthesis methods, (**b**) SEM image, (**c**) TEM image, and (**d**) adsorption reaction. Reprinted with permission from Ref. [94], Copyright 2016, Elsevier Ltd. (**e**,**i**) shell-core MgH_2_@CA microspheres for the adsorption of H_2_: (**e**) synthesis method, (**f**) SEM image, (**g**) adsorption capacity, (**h**) recyclability, and (**i**) adsorption and desorption at different temperatures. Reprinted with permission from Ref. [96], Copyright 2018, Elsevier Ltd.

Singh and co-workers demonstrated the synthesis of Pt-modified CAs through sol-gel formation of RF wet gel, ambient drying, and high-temperature pyrolysis [94]. As shown in Figure 10a, the addition of Na_2_CO_3_ into the mixed resorcinol and formaldehyde system induced molecular cross-linking and the formation of RF gel, which was then dried into RF aerogels at ambient pressure conditions. After high-temperature (1050 °C) treatment and cooling, the formed RF aerogels were successfully transferred into CAs. The formed RF aerogels exhibited a pearl-like particle structure, based on the obtained SEM image (Figure 10b), and the corresponding TEM image (Figure 10c), which indicated that the RF aerogels had a microporous structure with pore size of 0.30–1.46 nm, suitable for the filling and adsorption of H_2_ molecules. However, this is not considered a favorite for the filling of N_2_ molecules, as illustrated in Figure 10d. Finally, they found that the fabricated CAs exhibited a hydrogen storage capacity of about 5.65 wt.% at liquid N_2_ temperature, which was a little bit higher than that of the Pt-doped CAs (5.15 wt.%). Therefore, they suggested the special structure of CAs is crucial for the storage of H_2_, although the doping of Pt nanoparticles did not really contribute to the enhancement of H_2_ adsorption. In another case, Zhong et al. reported the solution-phase synthesis of Pd-doped CAs for the storage of H_2_ [95]. At <10 bar pressure region and 298 K, the H_2_ uptake capacity of CAs increased with the loading content of Pd nanoparticles. However, at higher pressure, the adsorption of H_2_ was affected, mainly by the pore volume of the fabricated CAs.

Peng et al. reported the synthesis of shell-core MgH_2_@CA microspheres for high-performance H_2_ storage [96]. As indicated in Figure 10e, formaldehyde and resorcinol were mixed together to form RF aerogels after CO_2_ supercritical drying at room temperature. After high-temperature pyrolysis at Ar flow, CAs with a microsphere structure were synthesized. After that, the ball-milling technique was utilized for the preparation of Mg@CA microspheres, which were further utilized to form MgH_2_@CA microspheres via a hydrogenation process. The synthesized MgH_2_@CA microspheres had an average size of 10 µm, in which small MgH_2_ particles were found to cover the surface of the CA microspheres (Figure 10f). The formed MgH_2_@CA microspheres revealed enhanced H_2_ adsorption compared to Mg power, MgH_2_, and mixed MgH_2_-CA (Figure 10g), ascribed to strong binding between Mg and H atoms, as well as the inhibited agglomeration by CA microspheres. In addition, the MgH_2_@CA microspheres exhibited very good cycle stability for the adsorption of H_2_ and high performance for adsorbing and releasing H_2_ at different temperatures (Figure 10h,i). This study provides a new strategy for the design and synthesis of functional hydrogen storage materials with good stability and high cycle ability. In a similar study, Utke and co-workers reported the fabrication of CA with 2LiBH_4_-MgH_2_ composites, which were then further modified with ZrCl_4_ for the fabrication of functional CMBAs [97]. After the modification with ZrCl_4_, the dehydrogenation temperature was decreased and the H_2_ storage capacity of the designed CMBAs was improved. The obtained results indicated more than 90% H_2_ storage capacity could be released and reproduced after 4 cycles of testing.

Graphene-based composite aerogels can also be used for H_2_ adsorption. For instance, Ren et al. demonstrated the synthesis of metal-organic framework (MOF)-functionalized graphene aerogel (GA) at mild reaction condition [98]. The increased H_2_ adsorption of the GA-MOF composites was ascribed to the increased GO layer number in GA, enhanced surface area by 2D MOF and GO materials, as well as increased pore volume.

In addition to the contributions of the selected materials for H_2_ adsorption, both experimental and theoretical studies have indicated that other factors. such as the temperature, and pressure, as well as the size, density, and active surface area of the aerogels, are also important for affecting the adsorption capacity of CMBAs.

### 4.4. Adsortion of Volatile Organic Compound Gases

With the wide use of cars and various vehicles, a lot of organic gases, also known as volatile organic compounds (VOCs), are produced, which cause great quantities of pollutants to enter the atmospheric environment. CMBAs exhibit high performance for the adsorption and removal of VOCs, including formaldehyde, toluene, benzene, xylene, and others. due to their properties of high adsorption capacity, good selectivity, high sustainability, and excellent reproducibility.

**Figure 11:**
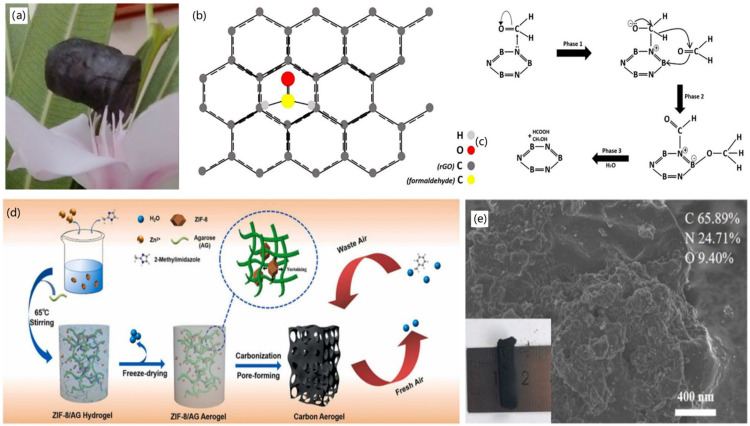
CMBAs for organic gas adsorption. (**a**–**c**) graphene aerogels for the adsorption of formaldehyde: (**a**) Photograph of rGO-hBN hybrid aerogels, (**b**) adsorption configuration of formaldehyde on rGO, and (**c**) adsorption reaction mechanism. Printed with permission from Ref. [99], Copyright 2021, Nature Publishing Group. (**d**,**e**) 3D porous CAs for the adsorption of toluene: (**d**) fabrication of CAs and the adsorption mechanism, and (**e**) SEM image of CAs, the inset is optical photo of CAs. Reproduced with permission form Ref. [103], Copyright 2021, Elsevier Ltd.

Androulidakis and co-workers reported the synthesis of multifunctional graphene-based aerogels by combining two types of 2D materials, rGO and *h*-BN [99]. To fabricate the hybrid CMBAs, rGO and *h*-BN were mixed with various volume ratios using the freeze-drying technique, by which hybrid GAs with ultralight mass and high mechanical properties were produced, as shown in Figure 11a. The fabricated hybrid CMBAs exhibited 7 times enhanced adsorption capacity of formaldehyde, compared with pure rGO aerogels, ascribed to a few advantages of the designed CMBAs. Firstly, the designed CMBAs showed highly specific surface area and a hydrophilic surface, improving the adsorption of polar formaldehyde molecules. Secondly, rGO nanosheets preferred to change the molecular structure of formaldehyde when it was adsorbed onto the neat sheets of graphene, as shown in Figure 11b. Thirdly, *h*-BN revealed high adsorption ability towards formaldehyde molecules, due to the existence of abundant hydroxyls and amines. In addition, the *h*-BN sheets induced highly effective chemical adsorption to formaldehyde molecules via the Cannizzaro-type disproportionation reaction (Figure 11c). Beside the adsorption of VOCs, the designed CMBAs had excellent water adsorption, high mechanical and thermal conductivity, due to the combination of 2D materials, and showed potential applications for protecting artefacts in high humidity and VOCs.

CMBAs can also be utilized for the adsorption of benzene and toluene gases. For instance, Rastegar et al. demonstrated the synthesis of a novel carbon xerogel through polymerization to form organic xerogel, with subsequent high temperature carbonization [100]. After the activation of NH_4_Cl, the formed carbon xerogel exhibited higher active surface area and benzene adsorption capacity than both organic and carbon xerogels. In another study, they prepared alumina-doped carbon aerogel (C/Al_2_O_3_) nanocomposites, which showed quick and effective adsorption of benzene from the flow gas in a fixed bed reactor [101]. Peng et al. reported the optimal fabrication of monolithic carbon foam via a simple templated synthesis method [102]. The combination of carbon foam with active carbon promoted the formation of a high-performance adsorbent of benzene with an adsorption capacity of 4692 mg/g.

For the adsorption of toluene gas, Zhang and co-workers reported the synthesis of a novel MOF-derived 3D CA with porous structure [103]. As shown in Figure 11d, ZIF-8 nanoparticles were first synthesized and then mixed with agarose (AG) for the synthesis of ZIF-8/AG hydrogel, which was then transferred into ZIF-8/AG aerogel after freeze-drying. After subsequent carbonization, CA with a porous structure was created, and typical SEM image and optical photograph of ZIF-8-derived CA are shown in Figure 11e. It was found that 2D layered AG was formed, which had large specific surface area, an abundant microporous structure, and high N content, which promoted the adsorption capacity towards toluene with 511.2 mg/g under 50% humidity. Due to the utilization of MOF materials for the design of CAs, this study provides a facile strategy for the regulation of the porous structure and surface functions for enhanced adsorption of VOCs with high selectivity and recyclability. Through the use of 2D graphene, Li and co-workers reported the synthesis of GAs through the gelation of GO with tetraethyl orthosilicate (TEOS) [104]. It was found that the addition of TEOS not only reduced the pore diameter and increased the specific surface area of GAs, but also improved the hydrophobicity of the formed Gas, therefore, enhancing adsorption ability towards both benzene and toluene gases.

CMBAs can also be used for the adsorption of VOCs from both dry and wet air. For instance, highly effective adsorption and removal of benzene, toluene, and xylenes was previously achieved by applying gallic acid-resorcinol-based CAs [105] and monolithic CAs [106].

## 5. Conclusions and Perspectives

In summary, we have presented the progress of the synthesis, functional regulation, and gas adsorption applications of CMBAs. From the above introduction and discussion, it can be concluded that various CMBAs can be synthesized effectively through templated synthesis, hydrothermal synthesis, self-assembly, 3D printing, and others. For the formation of CMBAs, in some cases gelation, via chemical and physical cross-linking, freeze-drying, and carbonization are necessary. Through adjusting the porosity and periodicity of CMBAs, the structure of the synthesized aerogels can be tailored. In addition, the functions of CMBAs can be enhanced via surface chemical modification and hybridization with other nanomaterials, such as nanoparticles, quantum dots, nanowires, and 2D materials. Therefore, by structural and functional regulations, various CMBAs from different carbon precursors, including polymer-derived carbon, CNTs, CNFs, graphene, and carbides, have been successfully fabricated. The produced functional CMBAs reveal high potential for gas adsorption applications, due to their unique properties, such as having a hierarchical porous structure, interconnected nano-network, high specific surface area, and high porosity. case studies of CMBAs towards the adsorption and removal of CO_2_, CO, H_2_S, Hg vapor, H_2_, and organic gases were carried out. In the laboratory tests, the fabricated CMBAs exhibited great performance for the adsorption and removal of these gases. However, the preparation of real products for practical applications is limited. The challenges are mainly high cost, high energy consumption, and low sustainability of CMBAs, compared to other non-carbon adsorbents. We believe this review is useful for promoting the design and synthesis of functional CMBAs for various applications through tailoring the structure and functions of aerogels.

Here we would like to provide some perspectives for this promising research topic. Firstly, it is necessary to develop simple and economic methods for the synthesis of CMBAs; for instance, by decreasing energy consumption and utilizing some cheap precursor materials, such as biomass materials. In addition, new drying methods for carbon material hydrogels/3D nanostructures that do not destroy their network porous structure are highly needed. Secondly, the synthesis of carbide-based aerogels could be further studied, as carbide materials are currently widely used for environmental science and energy storage applications. The combination of carbides with polymers, nanoparticles, and biomolecules is beneficial for improving the functions of carbide-based aerogels. Thirdly, the sustainable applications of CMBAs should be further studied. To enhance the sustainability of CMBAs, precursor materials with degradable, regenerated, stable, and producible properties should be applied. Fourthly, theoretical simulations and adsorption mechanism analysis of CMBAs towards the adsorption of gas molecules could be investigated in-depth, which is crucial for understanding the interactions between materials and gas molecules and providing potential ideas for enhancing the functions of CMBAs and improving adsorption efficiency. Fifthly, practical applications of CMBAs should be further explored; for instance, the utilization of CMBAs for architectural decoration and as gas sensors.

## Figures and Tables

**Table 1:** Summary of the materials, structure, properties, and applications of CMBAs.

Type of CMBA	Materials	Synthesis Method	Structure/Properties	Application	Ref.
Polymer-carbon	PSDVB NPs	Polymerization and carbonization	Powdered CAs with high specific surface area	Energy storage	[41]
	Urea and lanthanum	Gelation and carbonization	Hole-like porous structure, hierarchical porous network	Environmental and energy science	[42]
	MXene and BC	Freeze-drying and carbonization	Parallel and continuous flake structure	Wearable devices	[43]
	Waste paper	Gelation and freeze-drying	Lightweight, hydrophobic and porous	Adsorbent of organics	[44]
	TOCN and MF	Coating and pyrolysis	High N content, high compressibility, high conductivity	Sensors and electrodes	[45]
	NFC	Gelation and freeze-drying	High porosity andelasticity, good hydrophobicity and lipophilicity	Adsorbents of oils and organic solvents	[46]
CNTs	CNT and PVA	Chemical linking and freeze-drying	Dandelion-like structure	Adsorbents of organic solvents	[47]
	MWCNTs and BC	Supercritical CO_2_ drying	Piezoresistive behavior	Strain sensor	[48]
	CNT and CMC	Freeze-drying	Low bulk density, high strength, good processability	Devices, catalysis	[49]
	CNT and Pd NPs	Dry-spinning and thermal evaporation	Porous structure with Pd doping	H_2_ sensing	[50]
	SWCNTs and h-BN	Coating and pyrolysis	Ultra-compressivity, high porosity, excellent elasticity	Ceremic materials	[51]
	CMPA	Polymerization and carbonization	Hierarchical nanoporous structure; hollow nanotube structure	Solar steam generation	[6]
	Toluene	FC-CVD	Lightweight, hydrophobic, and porous	Oil adsorption	[52]
	Methenol	FC-CVD	Highly porous and ultralight	Liquid adsorption and energy storage	[53]
CNFs	BC nanofibers	Freeze-drying and carbonization	Hierarchical honeycomb cellular structure	Nanodevices	[56]
	Chitin nanofibers	Freeze-drying and carbonization	Homogeneous nanofiber structure	Dye adsorption and energy storage	[57]
	NFC	Freeze-drying and carbonization	Stacked nanosheet structure, good electrical conductivity, and high compressivity	Supercapacitors, absorbents	[58]
	PAN and ZIF-8	Electrospinning, freeze-drying, and carbonization	Cellular framework, low density, elastic	Adsorption of ions, electrocatalysis, energy storage	[59]
	BC and MoC	Freeze-drying and carbonization	3D network	Desalination	[60]
Graphene	GO, PVA, vitamin C, and AANi	Cooling and carbonization	Magnetic 3D GA	Microwave absorption	[27]
	GO and silica filler	3D printing, freeze-dryging, carbonization	Cubic lattice consisting of multiple parallel cylindrical filament	supercapacitors	[63]
	GO	3D printing, freeze-dryging, carbonization	3D honeycomb-like porous structure	supercapacitors	[64]
	Melamine foram and GO	Coating and drying	Ordered porous structure	Adsorption of organic solvents	[65]
	GO and borate	Hydrothermal synthesis, freeze-drying	Reversible compressibility, high conductivity, and low thermal conductivity	Pollution prevention and nanodevices	[66]
Carbides	CF/SiO_2_	Thermal reduction and calcination	Porous network	Thermal insulation	[67]
	CA and TiC/NbC	Freeze-drying, high temperature reaction	Higher specific surface area and lower bulk density	electrocatalysis	[68]
	RF/SiO_2_	Thermal reduction and supercritical CO_2_ drying	Mesoporous	Energy devices	[69]
	GO and SiC nanowires	Freeze-casting and carbothermal reducation	Whole X-band attenuation	Electromagnetic wave attenuation	[70]
	Silk fibers and SiC nanowires	In-situ growth and carbothermal reduction	Highly porous, ultralight density, and good compression resistance	Microwave attenuation and thermal insulator	[71]

## Data Availability

Not applicable.
